# Rivaroxaban versus standard anticoagulation for acute venous thromboembolism in childhood. Design of the EINSTEIN-Jr phase III study

**DOI:** 10.1186/s12959-018-0188-y

**Published:** 2018-12-21

**Authors:** Anthonie W. A. Lensing, Christoph Male, Guy Young, Dagmar Kubitza, Gili Kenet, M. Patricia Massicotte, Anthony Chan, Angelo C. Molinari, Ulrike Nowak-Goettl, Ákos F. Pap, Ivet Adalbo, William T. Smith, Amy Mason, Kirstin Thelen, Scott D. Berkowitz, Mark Crowther, Stephan Schmidt, Victoria Price, Martin H. Prins, Paul Monagle

**Affiliations:** 1 Bayer AG, Research and Development, Thrombosis and Hematology, Building 402, room 304, Aprather Weg 18a, 42113 Wuppertal, Germany; 2 Department of Paediatrics, Medical University of Vienna, Wien, Austria; 3 Children’s Hospital Los Angeles, Los Angeles, USA; 4 Sheba Medical Center, TelHashomer, Israel; 5 University of Alberta, Edmonton, Canada; 6 Department of Pediatrics, McMaster University, Hamilton, Canada; 7 Giannina Gaslini Children’s Hospital, Genoa, Italy; 8 University Hospital Schleswig-Holstein, Kiel, Germany; 9 Department of Medicine, Hematology and Thromboembolism McMaster University, Hamilton, ON Canada; 10 Center for Pharmacometrics & Systems Pharmacology, Department of Pharmaceutics University of Florida, Orlando, FL USA; 11 Department of Pediatrics, Division of Pediatric Hematology-Oncology Dalhousie University, Halifax, Canada; 12 Maastricht University Medical Center (MUMC+), Maastricht, The Netherlands; 13 Royal Children’s Hospital, University of Melbourne, Murdoch Childrens Research Institute, Melbourne, Australia

**Keywords:** Anticoagulation, Bodyweight-adjusted dosing, Pediatric patients, Rivaroxaban, Venous thromboembolism

## Abstract

**Background:**

Venous thromboembolism (VTE) is a relatively rare condition in childhood with treatment mainly based on extrapolation from studies in adults. Therefore, clinical trials of anticoagulation in children require novel approaches to deal with numerous challenges. The EINSTEIN-Jr program identified pediatric rivaroxaban regimens commencing with in vitro dose finding studies followed by evaluation of children of different ages through phase I and II studies using extensive modeling to determine bodyweight-related doses. Use of this approach resulted in drug exposure similar to that observed in young adults treated with rivaroxaban 20 mg once-daily.

**Methods:**

EINSTEIN-Jr phase III is a randomized, open-label, study comparing the efficacy and safety of rivaroxaban 20 mg-equivalent dose regimens with those of standard anticoagulation for the treatment of any types of acute VTE in children aged 0–18 years.

A total of approximately 500 children are expected to be included during the 4-year study window. Flexibility of treatment duration is allowed with study treatment to be given for 3 months with the option to continue treatment in 3-month increments, up to a total of 12 months. However, based on most common current practice, children younger than 2 years with catheter-related thrombosis will have a main treatment period of 1 month with the option to prolong treatment in 1-month increments, up to a total of 3 months.

**Conclusions:**

EINSTEIN-Jr will compare previously established 20 mg-equivalent rivaroxaban dosing regimens with standard anticoagulation for the treatment of VTE in children. Demonstration of similarity of disease, as well as equivalent rivaroxaban exposure and exposure-response will enable extrapolation of efficacy from adult trials, which is critical given the challenges of enrollment in pediatric anticoagulation trials.

**Trial registration:**

Clinicaltrials.gov NCT02234843, registered on 9 September 2014.

## Background

Venous thromboembolism (VTE) is a common condition in adults [1]. Anticoagulants are the mainstay of treatment of VTE and guidelines recommend therapy for three months or longer [2]. In children, VTE is relatively rare with reported incidences being approximately 100 times lower than in adults [3–8].

Pediatric clinical practice guidelines for anticoagulant treatment in VTE are mainly based on extrapolation from trials in adults [2]. The standard practice in pediatric VTE is to monitor anticoagulant therapy within target therapeutic ranges, extrapolated from adult studies [2, 9], which is made difficult due to the challenges of injections and collecting blood samples in children. Vitamin K antagonist (VKA) therapy poses further challenges due to interactions with foods and medications, lack of age-specific liquid preparations and poor compliance, rendering anticoagulation management of children logistically speaking problematic. The availability of an oral anticoagulant treatment for children that does not require subcutaneous or intravenous injections and regular laboratory monitoring, which is now standard of care in adults [10–12], is desirable.

Rivaroxaban is an oral, direct factor Xa inhibitor. For treatment of acute VTE in adults, rivaroxaban is given at a dose of 15 mg twice-daily for 21 days, followed by 20 mg once-daily thereafter [10–12]. This regimen is as effective as conventional therapy (consisting of a parenteral anticoagulant followed by a VKA) and is associated with fewer major bleeding events and improved treatment satisfaction [10–14].

Given that no previous randomized trial of anticoagulation in children has ever successfully completed target enrollments or could be powered for efficacy [15–18], novel approaches are required to successfully evaluate rivaroxaban for the treatment of VTE in childhood. In collaboration with the European Medicines Agency and the US Food and Drug Administration of the United States, pediatric development plans were set up to evaluate rivaroxaban for the treatment of VTE, including development of age-appropriate formulations [19, 20]. First, the anticoagulant effect of rivaroxaban in children and neonates was evaluated in vitro [21, 22]. Then, a physiologically-based pharmacokinetic (PBPK) model was developed to inform the dosing regimen for clinical studies in children [23]. Subsequently, in the EINSTEIN-Jr Phase I and II studies [24–28], we established body weight-adjusted rivaroxaban regimens for children aged birth to 18 years that will result in exposures as those observed in young adults treated with the therapeutic dose of rivaroxaban 20 mg once-daily. In these pediatric studies, rivaroxaban was well-tolerated, with no major bleeding or recurrent VTE and low rates of clinically relevant non-major bleeding [26–28]. In addition, comparisons of post-treatment with pre-treatment imaging tests showed that rivaroxaban was associated with a reduced clot burden in most evaluated children.

To optimize recruitment of children with clinically significant VTE requiring anticoagulant therapy in EINSTEIN-Jr. phase III, multiple pragmatic decisions were made regarding selection of the inception cohort, application of exclusion criteria, and allowance of flexible durations of study medication. To optimize extrapolation of efficacy from adults with VTE who were treated with rivaroxaban in phase II and III studies [10, 11, 29], identical criteria for symptomatic and surrogate efficacy outcomes and for bleeding events were selected.

The purpose of the EINSTEIN-Jr Phase III study is to compare the efficacy and safety of the pediatric rivaroxaban regimens with standard anticoagulation for the acute and continued treatment of VTE in children.

## Methods

### Study objectives

The main efficacy objective is to document the efficacy of rivaroxaban regimens at a 20 mg-equivalent dose for the prevention of fatal or symptomatic non-fatal recurrent VTE. The secondary efficacy objective is to assess the incidence of symptomatic recurrent VTE and asymptomatic deterioration on repeat imaging. The principal safety objectives are to document the incidence of major and clinically relevant non-major bleeding [30, 31]. An additional objective is to characterize the pharmacokinetic and pharmacodynamic profile of rivaroxaban.

### Study design and oversight

The EINSTEIN-Jr study is a multicenter, randomized, open-label, active-controlled study comparing the efficacy and safety of rivaroxaban regimens in a 20 mg-equivalent dose with those of standard anticoagulation for the treatment of acute VTE in children. The trial sponsors are Bayer AG and Janssen Research & Development, LLC. The steering committee, which is comprised of academic and sponsor members, has final responsibility for the design of the trial, the development of the protocol and oversight of the study. The protocol will be approved by the Institutional Review Board or Ethics Committee of each participating center. Written permission from a parent or guardian and, when appropriate, child assent, is obtained. An independent data monitoring committee will periodically review the study outcomes and provide recommendations to the steering committee. A central, blinded, independent adjudication committee will evaluate all index and suspected recurrent VTE events, deaths, and all suspected episodes of bleeding. In addition, the committee will review all repeat imaging tests performed at the end of the main study treatment period. A 24-h medical emergency line will be available throughout the study. The study is registered at clinicaltrials.gov (NCT02234843).

### Patients

Children will be enrolled from approximately 125 sites in 30 countries over a time window of approximately 4 years. The first child was randomized in November 2014, and the last visit of the last child to be included is projected for the first quarter of 2019. The study is expected to include a total of approximately 500 children. Children aged 0–18 years are eligible for inclusion if they have objectively confirmed acute VTE. Children are ineligible if they have active bleeding or high risk for bleeding contraindicating anticoagulant therapy (Table 1). Additional ineligibility criteria include hepatic disease associated with a coagulopathy and an estimated glomerular filtration rate < 30 mL/min/1.73 m^2^ [32]. The full list of eligibility criteria is provided in Table 1. Children with active cancer are also potentially eligible for the study [33]. Non-steroid anti-inflammatory drugs and antiplatelet agents are strongly discouraged since they increase the risk for bleeding in patients treated with anticoagulants [34]. However, if such medication is indicated, the lowest possible dose should be selected.

**Table 1:** Inclusion and exclusion criteria

Inclusion	
1. Children aged 0 months to < 18 years with confirmed venous thromboembolism who receive initial treatment with therapeutic dosages of unfractionated heparin, low molecular weight heparin or fondaparinux and require anticoagulant therapy for at least 90 days. However, children aged 0 months to < 2 years with catheter-related thrombosis require anticoagulant therapy for at least 30 days. 2. Informed consent provided and, if applicable, child assent provided. 3. Children younger than 6 months need to have a gestational age at birth of at least 37 weeks, oral feeding/nasogastric/gastric feeding for at least 10 days, and body weight ≥ 2600 g.	
Exclusion	
1. Active bleeding or high risk of bleeding contraindicating anticoagulant therapy. 2. An estimated glomerular filtration rate < 30 mL/min/1.73 m^2^ (in children < 1 year: serum creatinine 97.5th percentile). 3. Hepatic disease which is associated with either: coagulopathy leading to a clinically relevant bleeding risk, or alanine aminotransferase > 5× ULN or total bilirubin > 2× ULN with direct bilirubin > 20% of the total. 4. Platelet count < 50 × 10^9^/L. 5. Hypertension defined as systolic and/or diastolic blood pressure > 95th age percentile. 6. Life expectancy < 3 months. 7. Concomitant use of strong inhibitors of both cytochrome P450 isoenzyme 3A4 and P-glycoprotein, i.e. all human immunodeficiency virus protease inhibitors and the following azole-antimycotics agents: ketoconazole, itraconazole, voriconazole, posaconazole, if used systemically (fluconazole is allowed). 8. Concomitant use of strong inducers of cytochrome P450 isoenzyme 3A4, i.e. rifampicin, rifabutin, phenobarbital, phenytoin and carbamazepine. 9. Childbearing potential without proper contraceptive measures, pregnancy or breast feeding. 10. Hypersensitivity or any other contraindication listed in the local labeling for the comparator treatment or experimental treatment. 11. Inability to cooperate with the study procedures. 12. Previous assignment to treatment during this study. 13. Participation in a study with an investigational drug or medical device within 30 days prior to randomization.	

ULN, upper level of normal

The study will start with the evaluation of children aged 12–18 years since the rivaroxaban body weight-adjusted dosing regimen has been confirmed for this age group in phase II studies and was approved by the data monitoring committee and steering committee. Once the body weight-adjusted dosing regimen has been confirmed for children aged 6–12 years in phase II and has been approved by the data monitoring committee and steering committee, recruitment will be opened for this age group. This procedure will be repeated subsequently for children in the age groups 2–6 years, and birth–2 years.

Due to current limitations and uncertainties in pharmacokinetic (PK) predictions based on the PBPK modelling approach, children younger than 6 months need to have a gestational age at birth of at least 37 weeks, enteral feeding for at least 10 days, and a body weight of ≥2600 g.

### Randomization and stratification

Allocation to treatment will be done centrally by an interactive voice/web response system and will be done in a 2 (rivaroxaban) to 1 (standard anticoagulation) ratio. Allocation will be stratified by baseline presentation of VTE for each age group, separately, i.e. thrombosis of a) lower extremity, caval vein, right side of the heart, lungs, subclavian vein, upper extremity, or catheter-related thrombosis, and b) cerebral vein and sinuses, jugular vein, mesenteric vein, portal vein, or renal vein. Randomization can be done during the first 9 days of initial treatment.

### Study medication

Children randomized to rivaroxaban will start rivaroxaban the day following the last administration of initial therapy with unfractionated heparin (UFH), low molecular weight heparin (LMWH), or fondaparinux but with a minimal duration of initial therapy of 5 days and a maximum duration of 9 days. Children randomized to standard anticoagulation can continue UFH, LMWH, or fondaparinux or can switch to VKA therapy (Table 2) [2]. If VKA therapy is planned, it can be initiated any time after randomization. Initial therapy with UFH, LMWH, or fondaparinux can be stopped after a minimum of 5 days and only if the international normalized ratio is above 2 on two separate occasions, 24 h apart.

**Table 2:** Recommended regimens for treatment with standard anticoagulation in children [2]

Anticoagulant drug	Age or body weight	Initial dosage	Target
Warfarin			INR 2.0–3.0
Acenocoumarol			INR 2.0–3.0
Phenprocoumon			INR 2.0–3.0
Unfractionated heparin			aPTT times 1.5–2.5
Enoxaparin	< 2 months	1.5 mg/kg bid	
	> 2 months	1 mg/kg bid	
Tinzaparin	< 2 months	275 U/kg od	
	2–12 months	250 U/kg od	
	1–5 years	240 U/kg od	
	5–10 years	200 U/kg od	
	> 10 years	175 U/kg od	
Dalteparin		129 ± 43 U/kg od	
Reviparin	< 5 kg	150 U/kg bid	
	> 5 kg	100 U/kg bid	
Fondaparinux		0.1 mg/kg od	

*aPPT* activated partial thromboplastin time; *bid* twice-daily; *INR* international normalized ratio; *od* once-daily; *U* unit

Rivaroxaban will be administered in a body weight-adjusted 20 mg-equivalent total daily dose regimen using immediate release film-coated tablets with a dosage of 5, 10, 15, or 20 mg, or an oral suspension (1 mg/mL; Table 3) without laboratory-based dose adjustment. Children with body weight of ≥20 kg will receive rivaroxaban tablets or oral suspension. Children with body weight of < 20 kg will receive rivaroxaban as oral suspension. Rivaroxaban will be administered once-daily in children with a body weight of ≥30 kg. Children with a body weight below 30 kg required a substantially higher rivaroxaban dose (on a mg/kg basis) to achieve a similar exposure as compared to young adults treated with rivaroxaban 20 mg once-daily. Therefore, children with a body weight of 12–< 30 kg will have a twice-daily regimen, whereas those with a body weight < 12 kg will have a three times-daily regimen to avoid increased peak rivaroxaban plasma concentration*.* Dosing regimen, including dosing frequency, will be adjusted if the child’s body weight changes during the study. With a once-daily regimen, rivaroxaban will be taken in the morning during or within 2 h after breakfast. With a twice-daily regimen the morning dose will be taken during or within 1 h after breakfast and the evening dose will be taken during or within 1 h after dinner. With a thrice-daily regimen, the morning, afternoon and night doses will be taken during or within 30 min after feeding. Each rivaroxaban dose will be immediately followed by the intake of one typical serving of liquid up to 240 mL of liquid in older children or up to 120 mL in the younger ones.

**Table 3:** Body weight-adjusted rivaroxaban dosing schedule for children aged birth–< 18 years, as evaluated in EINSTEIN-Jr^a^

Body weight (kg)		Formulation	od dose	bid dose	tid dose	Total daily dose
Min	Max					
2.6	< 3	Oral suspension			0.8 mg	2.4 mg
3	< 4	Oral suspension			0.9 mg	2.7 mg
4	< 5	Oral suspension			1.4 mg	4.2 mg
5	< 6	Oral suspension			1.6 mg	4.8 mg
6	< 7	Oral suspension			1.6 mg	4.8 mg
7	< 8	Oral suspension			1.8 mg	5.4 mg
8	< 9	Oral suspension			2.4 mg	7.2 mg
9	< 10	Oral suspension			2.8 mg	8.4 mg
10	< 12	Oral suspension			3.0 mg	9 mg
12	< 20	Oral suspension		5 mg		10 mg
20	< 30	Tablet/oral suspension		5 mg		10 mg
30	< 50	Tablet/oral suspension	15 mg			15 mg
≥ 50		Tablet/oral suspension	20 mg			20 mg

^a^Dosing regimen, including dosing frequency, will be adjusted if the child’s body weight changes during the study. ^†^This dosing schedule may be subject to changes based on the results of the EINSTEIN-Jr phase III study; therefore, this dosing schedule cannot be used for the treatment of children with venous thrombosis outside the framework of the study

*bid* twice-daily, *od* once-daily, *tid* three-times-daily

### Study treatment duration

After randomization, children will receive either rivaroxaban or standard anticoagulation for a main study treatment period of 3 months except for children with catheter-related thrombosis aged younger than 2 years who have a main treatment period of 1 month. After the main study treatment period, the decision is made to stop study treatment or to continue. Study treatment can be continued for 3 blocks of 3 months each, with a maximum study treatment duration of 12 months. Children who turn 18 years after randomization can continue study treatment for the entire elected study treatment duration of 3, 6, 9, or 12 months. In children with catheter-related thrombosis younger than 2 years, study treatment can be continued for 2 blocks of 1 month each, with a maximum study treatment duration of 3 months.

### Risk factors for venous thrombosis at baseline

Using information recorded on the case report forms, the independent adjudication committee will evaluate and classify the reported risk factors for VTE (Table 4). Following the assessment of individual risk factors for VTE, VTE will be grouped as provoked by a) persisting risk factors; b) transient risk factors; c) persisting and transient risk factors; or d) unprovoked VTE [35]. Furthermore, the number of risk factors per individual child will be grouped as none, 1, or ≥ 2. In addition, for each child it will be assessed if the index event presented as a first or recurrent episode.

**Table 4:** Classification of risk factors for venous thrombosis at baseline

	Subgroup	Requirement
Provoked by persistent risk factor		
Active cancer – *n* (%)	• Hematologic malignancies • Solid tumors	Diagnosed, or treated within 3 months, or presence of metastases
Major organ disease – *n* (%)	• Cardiac • Gastrointestinal • Renal • Neurological	Exclude major infectious disease
Major congenital venous anomaly – *n* (%)		Venous anomaly in relation to location of index event
Known inherited thrombophilia – *n* (%) • Antithrombin, protein C or protein S deficiency • Factor V Leiden, or prothrombin mutation	• Homozygous • Heterozygous	At baseline or confirmed after randomization
Acquired thrombophilia – *n* (%)		Antiphospholipid syndrome. At baseline or confirmed after randomization
Family history of venous thrombosis – *n* (%)		First degree (parent or sibling)
Morbid obesity – *n* (%)		Body mass index > 35 kg/m^2^
Provoked by transient risk factor		
Major surgery – *n* (%)		Timeframe: within 1 month
Major trauma – *n* (%)		Timeframe: within 1 month
Major infectious disease – *n* (%)	• Systemic • Local	Timeframe: within 1 month Local infection in anatomical relation with index event
Prolonged immobilization – *n* (%)		Timeframe: within 1 month Duration immobilization > 1 week
Central venous catheter – *n* (%)		Timeframe: within 1 month Location of index event related to central venous catheter
Use of estrogens or progestins – *n* (%)		
Puerperium– *n* (%)		Puerperium ends 6 weeks after birth
Etiology of index venous thrombosis		
Unprovoked – *n* (%) Provoked by persistent risk factor – *n* (%) Provoked by transient risk factor – *n* (%) Provoked by persistent and transient risk factor – *n* (%)		
Number of risk factors – *n* (%) 0 1 ≥2		

### Repeat imaging

The repeat imaging test obtained at the end of the main treatment period will be compared with the baseline test and classified as normalized, improved (i.e., thrombus still present but partly recanalized or involving less venous segments), no relevant change (i.e., not recanalized and similar in extent) or deteriorated (i.e., new venous segment involved) [29].

### Surveillance and follow-up

All children will have regularly scheduled assessments in the clinic at days 30, 60, 90, and, if applicable days 180, 270, and 360. Children with suspected efficacy or safety outcomes will undergo appropriate diagnostic and/or laboratory testing. An observation visit is planned for all children 30 days after stopping study medication. All children who prematurely stop study treatment will be followed until the end of the intended treatment period. Continuation of anticoagulant therapy after study completion is at the discretion of the treating physician. Throughout the study, trained clinical research associates will conduct on-site monitoring visits and ensure quality review of data and effective interaction with study sites. During these visits, they will oversee data collection, review source documentation and case report forms, ensure regulatory compliance and resolve data queries. Adverse events will be described using the Medical Dictionary for Regulatory Activities.

### PK/PD assessments

The PK and pharmacodynamic (PD) effects of rivaroxaban will be described by the individual plasma concentrations, main PK parameters derived via population PK modeling (Area under the concentration time–curve from 0 to 24 h at steady state, maximum plasma concentration, and trough plasma concentration and the prothrombin time, activated partial thromboplastin time, and anti-factor Xa activity, respectively. In general, a sparse sampling approach will be applied to accommodate for the limited blood volume that can be taken in children. Blood sampling schemes for PK and PD depend on the dosing frequency and age: for once-daily and twice-daily treatments, PK and PD samples will be taken at four time intervals. For thrice-daily dosing, five PK samples and three PD samples will be taken at different days and time windows.

### Site training

Site physicians and their staff will be intensively trained for all aspects of the study by an online mandatory video training program, repeat investigator meetings and regular newsletters.

### Sample size considerations

A properly powered separate non-inferiority study in children with VTE is not feasible since accurate efficacy estimates in this population of standard anticoagulation versus either placebo, no treatment or less effective treatment are absent [36]. Thus, any formal calculation of the sample size is impossible. Moreover, a properly powered study based on the assumptions for trials in adults would require several thousands of children [10–12], which seems impossible due to the low incidence of VTE in childhood, the absence of a large pediatric network for thrombosis studies, and general challenges of enrolling children in clinical studies. Hence, the study has no formal sample size calculation. To provide the medical community with the best possible evidence, we have built a large network of sites specialized in care of children with acute VTE to enable enrollment of the highest possible number of children during the planned 4-years enrolment window. Consequently, we expect to be able to evaluate several hundreds of children with acute VTE during treatment with rivaroxaban or standard anticoagulation. As a consequence, recommendations for rivaroxaban treatment in children will have to be based on data from the EINSTEIN-Jr program and leverage of data obtained from rivaroxaban trials in adults with VTE.

To allow extrapolation of efficacy from adults to the pediatric population [20], the following conditions are considered. First, the pediatric 20 mg-equivalent rivaroxaban dose regimens should result in similar achieved drug exposure in children as observed in adults who received the approved therapeutic dose of 20 mg once-daily. Second, the clinical course of VTE (i.e. incidences of symptomatic recurrent VTE, major bleeding and mortality) should be similar in children and adults. Finally, the response to rivaroxaban therapy as compared to standard anticoagulation should be similar in children and adults. To document this similarity, two-sided 95, 90, 80, and 50% confidence intervals (CIs) will be calculated both for the individual incidences in the treatment arms and the relative effect estimate. As a reference, Table 5 specifies the 3-month incidences of recurrent VTE, major bleeding and mortality, as well as the 3-month relative efficacy and safety estimates, as observed in the EINSTEIN DVT/PE phase III program. These studies have randomized 8281 adult patients with acute VTE to either rivaroxaban or standard anticoagulation [10–12].

**Table 5:** Three-month recurrent VTE, bleeding, and mortality incidences in adult patients in EINSTEIN DVT and EINSTEIN PE [10–12]

	Rivaroxaban n/N (%; 95% CI)	Standard anticoagulation n/N (%; 95% CI)	Absolute risk difference (95% CI)	Hazard ratio (95% CI)
Recurrent VTE	69/4150 (1.7; 1.3–2.1)	82/4131 (2.0; 1.6–2.5)	0.3 (−0.3–0.9)	0.82 (0.60–1.13)
Major bleeding	28/4130 (0.7; 0.5–1.0)	49/4116 (1.2; 0.9–1.6)	0.5 (0.1–0.9)	0.55 (0.35–0.88)
Major and clinically relevant nonmajor bleeding	286/4130 (6.9; 6.2–7.7)	287/4116 (7.0; 6.2–7.8)	0.0 (−1.1–1.1)	0.98 (0.83–1.16)
Mortality	53/4150 (1.3; 1.0–1.7)	61/4131 (1.5; 1.1–1.9)	0.2 (−0.3–0.7)	0.81 (0.56–1.17)

Differences in incidences and their 2-sided 95% CIs were calculated by stratified Mantel–Haenszel method (strata: intended treatment duration and index event)

CI, confidence interval

### Statistical analysis

Efficacy outcomes will be considered for the entire study duration in all children who received at least one dose of study medication. Safety outcomes will be considered during study medication. Efficacy and safety results will be reported separately for the main study period (i.e. the initial 1-month period for children younger than 2 years who have catheter-related VTE and the initial 3-month period for all other children) and the period of continued treatment following completion of the main study period.

Analyses for recurrent VTE, and bleeding which occurred during the main treatment period will be done with a Cox proportional hazards model, stratified according to index diagnosis and age groups. Absolute differences in risk with 95% CIs will be calculated. Kaplan–Meier curves will be constructed to display the distribution of events over time. In addition, absolute risk differences and their 95% CIs will be calculated. The classification of the repeat imaging test at 3 months will be compared between the groups using a nonparametric test for stratified ordinal response data. The relationship of demographic and clinical characteristics, including anatomical location, etiology, and presence of residual VTE on repeat imaging, on the decision to extend anticoagulant therapy at the end of the main treatment period will be described.

## Discussion

In adults, the development of rivaroxaban therapy for the treatment of VTE followed the classical approach of large phase I and II studies to describe its pharmacokinetic and pharmacodynamic profile and to determine potential treatment regimens followed by adequately powered phase III studies to demonstrate non-inferiority versus standard anticoagulation [37]. In children, such a development program is notoriously difficult because of the low incidence of VTE in childhood and the many logistical hurdles to recruit children. Therefore, novel approaches which take advantages of the large body of evidence obtained in adults are required to identify efficacious and safe rivaroxaban dose regimens for the treatment of pediatric thrombosis [20].

The selection of the pediatric rivaroxaban dose regimens for EINSTEIN-Jr. phase III started with extrapolation of data of plasma concentrations obtained in adult VTE patients who were treated with rivaroxaban applying extensive PBPK modeling [23]. The dosing assumptions were followed by careful evaluation of PK, PD, safety, and efficacy of rivaroxaban treatment in children of different ages through phase I and II studies [26–28]. This resulted in bodyweight-related rivaroxaban tablet and oral suspension regimens that were associated with similar plasma concentrations as observed in young adults treated with rivaroxaban 20 mg.

EINSTEIN-Jr phase III will aim for the best possible inclusion rate of children with acute VTE during the 4-year study window but nevertheless will be underpowered for efficacy. Therefore, final acceptance of the selected rivaroxaban dose regimens will be depend on: 1) confirmation of equivalence in achieved exposure and exposure-response as compared to results obtained from young adults treated with rivaroxaban 20 mg; 2) demonstration of similarities between the clinical course of VTE in children and adults in terms of incidences of symptomatic recurrent VTE, major bleeding and mortality; and 3) qualitative analyses of the relative effect estimates of rivaroxaban therapy as compared to current standard anticoagulation practices in children and adults. In Table 6, the results with hypothetical hazard ratios are presented. The upper limit of the 95% CIs for symptomatic recurrences (main efficacy outcome) in case of equal outcome frequency is 3.72. For the secondary efficacy outcome (the combination of recurrences and asymptomatic deterioration on repeat imaging), the value of the upper margin of the 95% CI (i.e. 1.80) is well within the established range for non-inferiority of non-vitamin K antagonist oral anticoagulants versus standard of treatment in VTE [36]. It should be noted that worsening of a thrombus, even without clinical signs, is a clinically relevant observation. Because the study is not powered for non-inferiority, and a non-inferiority margin was not a priori specified, we will refrain from such conclusion in case the final results seem to favor rivaroxaban. However, non-inferiority of rivaroxaban to standard anticoagulation in children can be assumed if the upper margin of the CI around the hazard ratio is 2.5 or lower (i.e., preservation of 50% of treatment effect), 2.0 or lower (i.e., preservation of 66% of treatment effect), or 1.75 or lower (i.e., preservation of 75% of treatment effect) [37].

**Table 6:** Hypothetical 95% CIs around different rivaroxaban–standard anticoagulation hazard ratio assumptions

Assumed rivaroxaban–standard anticoagulation hazard ratio	Assumed 95% CI if incidence of the main efficacy outcome is 2.0% on standard anticoagulation^a^	Assumed 95% CI if incidence of the secondary efficacy outcome is 10% on standard anticoagulation^a^
0.500	0.11–2.28	0.25–0.99
0.667	0.16–2.76	0.35–1.26
0.800	0.20–3.14	0.43–1.48
0.930	0.25–3.52	0.51–1.69
1.000	0.27–3.72	0.56–1.80
1.075	0.29–3.94	0.60–1.92
1.250	0.35–4.45	0.71–2.21
1.500	0.43–5.18	0.86–2.61
2.000	0.60–6.64	1.17–3.42

Hypothetical 95% CIs around various hazard ratios assuming an incidence of 2.0% for the main efficacy outcome and 10% for the secondary efficacy outcome in the standard anticoagulation group in a population of 500 children randomized to rivaroxaban or standard anticoagulation in a 2 to 1 fashion. ^a^Based on approximation, CI, confidence intervals

To maximize the validity of a comparison of results of this study with previous results obtained with rivaroxaban in adults with VTE, the study will apply similar eligibility criteria, study duration and definitions of efficacy and safety outcomes [10–12]. However, to approach the challenge of recruitment with a rare disease, we aim to consider for inclusion a broad spectrum of children with VTE who would generally be considered for anticoagulant treatment. Thus, children can be considered for participation in the study if they have any manifestation of VTE requiring therapeutic anticoagulation, rather than VTE limited to the lower extremity or pulmonary embolism, which is the case in most anticoagulant studies in adults. In accordance with the rivaroxaban program in adults and to reflect what occurs in daily practice, we aimed to minimize the set of exclusion criteria as much as possible. Therefore, children are only deemed ineligible for the study if they have a condition contraindicating the use of standard anticoagulation, have a condition which meets regulatory contraindications for the use of rivaroxaban, or if they are likely to not complete the study as intended.

The minimum study treatment duration in our study is set at 3 months which is similar to the evidence-based treatment duration in adults with symptomatic VTE [38]. For those children who require longer treatment durations, the protocol allows for continued treatment durations with a maximum of three times three additional months. However, in neonates and young children, VTE is often a complication of indwelling catheters for which a 6-week to 3-month duration of anticoagulant therapy is suggested by international guidelines [2], albeit based on low grade evidence. To ensure that the protocol in EINSTEIN-Jr. will allow inclusion of neonates and children younger than 2 years with catheter-related VTE, the minimum study treatment duration is set at 1 month for these children [39]. For those cases where physicians deem longer treatment durations necessary, the protocol allows for continued treatment durations with a maximum of 2 times 1 additional month.

The main limitations of our study are the following. First, the study lacks a double-blind design which was chosen because of ethical concerns about the intolerability of placebo subcutaneous injections or intravenous infusion and mock laboratory testing in children. To minimize the potential for bias, investigators and co-investigators will be trained on the detection, documentation and reporting of potential study outcomes. Patient booklets, detailing signs, and symptoms of study outcomes and contact numbers, will be provided. Furthermore, children in both study arms will be seen in the hospital at regular time intervals, at which time the occurrence of potential study outcomes will be explored using standardized questionnaires. In case of suspected outcomes, a standardized diagnostic work-up will be performed and results will be communicated in an expeditious manner to the central adjudication office, which will evaluate adjudication dossiers for quality and completeness and will contact study sites in case of deficiencies. Moreover, reconciliation of the adverse event and study outcome databases will be performed to discover potential unreported study outcomes. Finally, an independent adjudication committee, whose members are unaware of the study-group assignment, will evaluate the initial diagnosis, all suspected outcomes and repeat thrombosis imaging tests at the end of the main treatment period. Second, the worldwide approved rivaroxaban regimen in adults, which does not require initial treatment with heparins [10–12, 40], cannot be applied in many children with VTE since most potentially eligible children will have already received therapeutic heparinization for several days before they can be considered for participation in our study. This is because of time involved to transfer children to tertiary centers and prolonged time involved in obtaining informed consent. To avoid large numbers of children being disqualified due to prolonged initial heparinization, we decided to adapt the rivaroxaban regimen by requiring at least 5 days of initial heparinization followed by rivaroxaban in a 20 mg-equivalent dose. We believe that this adaptation of the adult rivaroxaban regimen will not affect efficacy in children and considerably improve the generalizability of our findings to the broader pediatric VTE population. Third, children who will be allocated to standard anticoagulation will be allowed to be treated with locally accepted anticoagulation regimes to accommodate the wide array of preferences of participating pediatric hematologists [2].

Finally, restrictions with regards to birth weight and feeding may exclude some very young babies from participating in our study. This is because maturation of organs involved in rivaroxaban absorption and clearance depend on the gestational and postnatal age. Gastrointestinal conditions are more stable in children with a gestational age of ≥37 weeks who have been on oral feeding for at least 10 days. In addition, PK studies in very small children are restricted because of the limited sample volumes that are allowed to be obtained [41].

## Conclusions

In conclusion, this large pediatric phase III study will assess our previously established body weight-adjusted dosing regimen with rivaroxaban across the age spectrum of children who have thromboses of varied locations and who require acute and continued VTE treatment. Pediatric anticoagulation trials need to be flexible and innovative to provide useful data to help improve the anticoagulation therapy available to children.

## Abbreviations

aPTT: Activated partial thromboplastin time
bid: Twice-daily
CI: Confidence interval
DVT: Deep vein thrombosis
INR: International normalized ratio
LMWH: Low molecular weight heparin
od: once-Daily
PBPK: Physiologically based pharmacokinetic
PD: Pharmacodynamic
PE: Pulmonary embolism
PK: Pharmacokinetic
tid: Three-times-daily
UFH: Unfractionated heparin
VKA: Vitamin K antagonist
VTE: Venous thromboembolism
